# Fear of relapse in schizophrenia: a mixed-methods systematic review

**DOI:** 10.1007/s00127-022-02220-2

**Published:** 2022-02-13

**Authors:** Zofia Zukowska, Stephanie Allan, Emily Eisner, Li Ling, Andrew Gumley

**Affiliations:** 1 Institute of Health and Wellbeing, University of Glasgow, Glasgow, Scotland; 2 Division of Psychology and Mental Health, University of Manchester, Manchester, UK; 3 Greater Manchester Mental Health NHS Foundation Trust, Prestwich, UK

**Keywords:** fear of relapse, schizophrenia, psychosis, wellbeing

## Abstract

**Introduction:**

Fears of relapse in people diagnosed with schizophrenia have long been recognised as an impediment to recovery and wellbeing. However, the extent of the empirical basis for the fear of relapse concept is unclear. A systematic review is required to collate available evidence and define future research directions.

**Methods:**

A pre-registered systematic search (PROSPERO CRD42020196964) of four databases (PubMED, MEDLINE-Ovid, PsycINFO-Ovid, and Cochrane Central Register of Controlled Trials) was conducted from their inception to 05/04/2021.

**Results:**

We found nine eligible studies. Five were quantitative (4 descriptive and 1 randomised controlled trial), and four were qualitative. The available quantitative evidence suggests that fear of relapse may have concurrent positive relationships with depression (*r* = 0.72) and suicide ideation (*r* = 0.48), and negative relationship with self-esteem (*r* = 0.67). Qualitative synthesis suggests that fear of relapse is a complex phenomenon with behavioural and emotional components which has both direct and indirect effects on wellbeing.

**Conclusions:**

Evidence in this area is limited and research with explicit service user and carer involvement is urgently needed to develop new and/or refine existing measurement tools, and to measure wellbeing rather than psychopathology. Nonetheless, clinicians should be aware that fear of relapse exists and appears to be positively associated with depression and suicide ideation, and negatively associated with self-esteem. Fear of relapse can include fears of losing personal autonomy and/or social/occupational functioning. It appears to impact carers as well as those diagnosed with schizophrenia.

## Introduction

Schizophrenia is a severe mental health condition [1]. Following a first episode, many individuals recover [2], but relapse is common, with one recent estimate suggesting 46.4% relapse within 5 years of diagnosis [3]. Relapses are costly to health services [4]. For patients, relapse is associated with increased social isolation, decreased functionality [5, 6], and increased suicide risk [7]. Relapses also have profound negative impacts upon relationships with carers [8]. Antipsychotics have the best evidence for relapse prevention [9], but do not prevent relapse entirely [10]. Therefore, identification of early warning signs (EWS) of relapse is a cornerstone of relapse prevention within mental health services [11–13]. While relapse prevention is a major focus of clinical care [11], it is important to highlight that relapse is a contested construct with numerous definitions proposed [9, 14–16], a lack of consensus regarding definition [17], and an apparent lack of service user or carer involvement in determining relapse definition. Outcome concerns notwithstanding, existing literature suggests people with a schizophrenia diagnosis may dread relapse events. Potentially noted as far back as 1814 [18, 19], fear of relapse describes often devastating assumptions about relapse [20]. Fear of relapse is an independent predictor of future relapse [20], making it a potentially useful focus for intervention. The cognitive interpersonal model [21] hypothesises that fear of relapse drives feelings of anxiety and shame, triggered by traumatic memories of past psychotic experiences (both from the psychosis itself and from the iatrogenic harm caused by psychiatric interventions). These feelings prompt patients to use defensive strategies to prevent relapse (e.g., hypervigilance or help-seeking) or to avoid re-traumatisation (e.g., avoiding help-seeking). Informal caregivers may have their own fears of relapse, making them important social actors within the cognitive interpersonal model. Despite longstanding observations that fear of relapse can have negative impacts upon wellbeing [22] (e.g., increased anxiety and depression), no review has yet summarised and conceptualised knowledge on this topic. While fear of relapse appears an important construct within relapse prevention and there is an existing measurement scale (Fear of Recurrence Scale, FoRSE) [20], it is unknown if this scale defines/assesses constructs that truly reflect how fear of relapse is experienced and understood by patients and carers. If a construct is not defined clearly, there is substantial ambiguity in what is being measured, creating potentially invalid inferences [23]. Beyond measurement ambiguity, there is a real risk of epistemic injustice [24] if patient and carer perspectives are not incorporated into the development of assessment tools. While the cognitive interpersonal model presents a conceptualisation of fear of relapse and includes carers, it would be improved by clearly mapping out the boundaries of what constitutes fear of relapse and by describing a clearer pathway for how experiencing fear of relapse impacts wellbeing. Acknowledging the lack of service user and carer involvement in relapse research more generally, this review aimed first to synthesise existing literature exploring fear of relapse and its impact on wellbeing from the perspective of patients and carers. A second key aim was to extend the existing theoretical framework by outlining further theoretical considerations derived from empirical research. To this end, this review addressed the following research questions:

1. In studies of fear of relapse, what clinical wellbeing outcomes have been measured and which of these are significantly associated with fear of relapse?
2. What do qualitative studies tell us about the meaning of fear of relapse for patients and/or informal caregivers?
3. In people with psychosis (and carers), what is the evidence that fear of relapse impacts wellbeing?

## Methods

### Overview

A systematic review and mixed-methods narrative synthesis were conducted, following a PROSPERO-registered (CRD42020196964) and PRISMA-compliant protocol [25].

### Study eligibility criteria

Studies were included if they: (1) explored fear of relapse in adults with schizophrenia spectrum diagnoses and/or their informal caregivers (aged 16 years and above), (2) explored the relationship between fear of relapse and wellbeing (broadly defined) using quantitative, qualitative, or mixed methodologies, and (3) were published in a peer-reviewed journal in English, French, or Polish. Studies were excluded if they: (1) explored fear of relapse in other mental health conditions (e.g., bipolar disorder) and (2) were not peer-reviewed.

### Information sources

Eligible studies were sourced in two stages: (1) four databases were searched from their inception to 05/04/2021: PubMED, MEDLINE-Ovid, PsycINFO-Ovid, and Cochrane Central Register of Controlled Trials (CENTRAL). A backward citation search of included articles was completed using Google Scholar.

### Search strategy

The search strategy was developed using database-specific search terms, with input from a clinical psychologist (AG) and someone with personal experience of schizophrenia relapse. Search logic and keywords were: (schizophrenia OR psychosis OR psychotic OR psychoses OR schizo*) AND (fear of relapse OR fear of recurrence OR post-traumatic stress OR post psychotic stress OR mental health anxiety).

### Selection process

Studies were manually and individually evaluated against the eligibility criteria by SA and ZZ.

### Data collection and data items

Data were exported to EndNote and extracted using a spreadsheet identifying: (a) authors and year of publication; (b) country/countries; (c) participant age (mean, standard deviation); (d) participant gender; (e) study design; (f) whether participants were patients, carers, or both, (g) diagnosis of participants (if relevant), and (h) wellbeing data. In qualitative studies, wellbeing data were quotations in which participants discussed fear of relapse in relation to their wellbeing. In quantitative studies, wellbeing data were mainly correlations between fear of relapse and depression. Data extraction was completed independently by ZZ and SA.

### Quality assessment and risk of bias

Study quality was assessed using the Mixed Methods Appraisal Tool (MMAT), which is designed for systematic reviews appraising mixed studies [26]. The MMAT assesses the quality of qualitative, quantitative, and mixed-methods research, as well as randomised and non-randomised controlled trials. SA and ZZ independently completed quality appraisal. Rating consensus was reached by discussion.

### Data synthesis and analysis

#### Overview of synthesis approach

In line with Hong and colleagues [27], this mixed-methods review followed a results-based convergent synthesis design. Qualitative and quantitative evidences were analysed separately using different synthesis methods, with the results of both syntheses integrated during a final narrative synthesis. We report the analysis methods in the order performed.

#### Quantitative analysis

Having appraised the mental health outcome measures in the data extraction table (Table 1), we concluded that there were not enough sufficiently homogenous outcomes to conduct a meta-analysis. Therefore, the limited dataset was synthesised using narrative synthesis, in line with synthesis without meta-analysis guidelines [28], by focussing on statistically significant correlations between fear of relapse and wellbeing measures.

**Table 1:** Study characteristics

Authors	Year	Country	Participants	Demographics	Study aim	Research design and methods	Fear of relapse assessment	Wellbeing assessment	Key summary	Quality (MMAT)
Baier	1995	USA	8 people diagnosed with schizophrenia, 5 carers	Age of participants with a diagnosis of schizophrenia: 48.8 (SD = 17.34) 73% female	To understand how persons living with chronic schizophrenia describe and live with the uncertainty of the illness	Semi-structured interviews	Qualitative	Qualitative	Uncertainty is a component of life with schizophrenia. The uncertainties concern the future, the medication, and the probability and time until relapse but they can also offer an opportunity for hope	3
Baker	1995	Canada	15 people diagnosed with schizophrenia	Age: 18 y/o—1; 20–29 y/o—2; 30–39 y/o—6; 40–49—4; 50–59—1; 70—1; 33% females	To identify how individuals witch schizophrenia detect early signs of relapse	Qualitative interview	Qualitative	Qualitative	Emotional distress was dominating participants' narratives. Participants actively fear relapsing. Immediate fears: losing equanimity, return of the distress related to their relapse, and impact of relapse on daily life	4
Collet et al.	2016	UK	16 people with persecutory delusions and a clinical diagnosis of non-affective psychosis	Age: 45.6 (SD = 12.1), 52% female	To explore correlates of wellbeing in people diagnosed with schizophrenia who experience persecutory delusions	Cross-sectional survey	MHWQ [33]	BDI [61], BSS [62], RSES [63]	MHWQ showed significant positive correlations with BDI and BSS and significant negative correlations with RSES	4
Sandhu	2013	UK	8 participants diagnosed with schizophrenia post-schizophrenic depression	Age: 25.4 (SD = 5), 38% females	To explore the post-psychotic depression in first episode psychosis and its phenomenological features	Qualitative interview and photo elicitation	Qualitative	Qualitative	Participants reported depression as linked to their experience of psychosis, the psychotic episodes were also leading to trauma, shame, doubt, and embarrassment	5
White and Gumley	2009	UK	27 people diagnosed with schizophrenia	Age: overall: 39.05; Non-PTSD group: 38.5 (10.7); PTSD group: 39.6 (10.3). Overall: 26% females; Non-PTSD group: 29% females; PTSD group: 20% females	To explore whether people with fear of relapse meet PTSD caseness	Quantitative descriptive	FoRSe	HADS [38], PP-PTSD (refers to unpublished source)	PTSD caseness was associated with fear of relapse, intolerance of uncertainty, and negative appraisals of paranoia. Fear of relapse was a significant predictor of PTSD caseness	4
Lal	2017	Canada	24 carers	Age: 49.6 (8.7), 75% female	To explore family perspectives on schizophrenia relapse in first-episode psychosis	Qualitative descriptive	Qualitative	Qualitative	The underlying theme in focus groups was worry, fear and anxiety concerning relapse related to the impact of the episode of psychosis, lack of confidence in coping skills and available clinical and emotional support	4
Gumley et al.	2015	UK	168 "relapse prone" people diagnosed with schizophrenia	Age: overall: 41.48; Group 1: 40.74 (11.33); Group 2: 42.22 (10.90); Overall: 28% females; Group 1: 23% females; Group 2: 34% females	To establish the reliability and validity of the Fear of Recurrence Scale as well as the sensitivity and specificity to relapse	Randomised control trial	FoRSe	CDS [64]	Significant positive correlation (*r* = 0.42) between FoRSe and CDS	4
Bassett, Sperlinger and Freeman	2009	UK	25 people diagnosed with persecutory delusions and a diagnosis of schizophrenia, schizoaffective disorder or delusional disorder or bipolar affective disorder or depression	Age: overall: 42.42; Group 1: 43.52 (13.57); Control group: 41.32 (12.28)	To investigate the presence of fear of madness in individuals with persecutory delusions and whether that fear is linked to paranoia	Quantitative non-randomized control trial	WAMH [33]	BAI [36], PSWQ [37]	Participants with persecutory delusions had higher levels of fear of madness than the control group on WAMH scales. Fear of madness was associated with higher levels of worry, anxiety, and distress	4
Herz and Melville	1980	USA	145 people with a diagnosis of schizophrenia; 80 relatives and carers	Age: overall: 38; Group 1: 38; Group 2: 38; Overall: 68% females; Group 1: 67% females; Group 2: 70% females	To explore the perception of early signs of relapse in individuals with schizophrenia and their relatives	Quantitative descriptive	Endorsing "fear of going crazy"	Relapse	Fear of going crazy in 28.3% of participants	4

#### Qualitative analysis

Thomas and Harden’s formal approach to meta-synthesis [29] was used. The approach consists of three stages: (1) line-by-line coding of extracted qualitative data (reported themes and researcher analysis) to allow for concepts to be ‘translated’ from one study to another; (2) establishing descriptive themes and developing new codes; (3) creating a final set of analytical themes. At all stages, ZZ and SA critically discussed the ongoing inductive analysis in detail.

#### Overall synthesis

Having analysed qualitative and quantitative data separately, results of both syntheses were integrated, creating a single summary exploring how fear of relapse relates to wellbeing. An in-depth, inductive thematic analysis of extracted data (including relationships between quantitative variables) was conducted, mapping common concepts across studies. ZZ completed an initial synthesis. Throughout analysis and development of the conceptual framework (of how fear of relapse is experienced), emerging ideas were discussed among the team.

#### Reflexivity

Due to the interpretivist nature of this review, which involved a team engaging with a variety of studies, we present a reflexivity section. This study was conducted within a critical realist paradigm [30], aiming to develop a theoretical framework focussing on the experiences of patients and carers while remaining mindful of what the researchers bring to this novel synthesis. The study design, including the search strategy, was developed jointly by (and as) a team who have direct expertise in psychosis and relapse (personal lived experience as service users, and/or professional experience as clinicians and researchers). This direct participatory involvement continued throughout the analysis and reporting of the results.

## Results

### Study selection

**Fig. 1:**
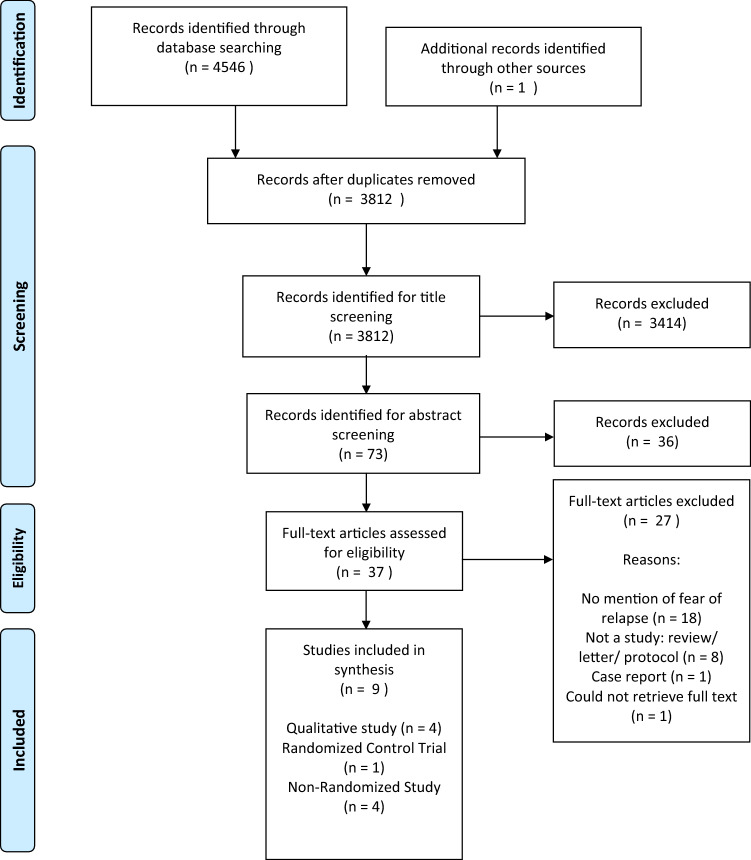
PRISMA flow diagram of the search

The initial search returned 4175 citations (3481 after de-duplication). Of these, 3411 were excluded after screening titles against inclusion/exclusion criteria and a further 33 after abstract screening. The remaining 37 full-text articles were read by ZZ and SA, who independently assessed final eligibility against inclusion/exclusion criteria. AG was asked to adjudicate on two disagreements. This process left eight studies included in overall synthesis, with one further study identified via backwards citation searching. From the database and hand searching, nine studies met inclusion criteria. Figure 1 shows the PRISMA flow diagram.

### Study characteristics

The selected studies [20, 31–34, 39–42] were conducted in the UK (*n* = 5), USA (*n* = 2), and Canada (*n* = 2). Further characteristics can be found in Table 1.

### Risk of bias

The mean quality rating of papers using the MMAT was 4**** (SD = 0.5) out of a possible 5****. A full assessment of methodological quality is provided in Table 2.

**Table 2:** Quality assessment of studies using MMAT

Authors	Year	Screening questions		Methodology																			
				Qualitative					Quantitative RCT					Quantitative non-RCT					Quantitative descriptive				
		Q1	Q2	Q1	Q2	Q3	Q4	Q5	Q1	Q2	Q3	Q4	Q5	Q1	Q2	Q3	Q4	Q5	Q1	Q2	Q3	Q4	Q5
Baier M	1995	Y	Y	Y	Y	Y	N	N															
Lal S et al.	2017	Y	Y	Y	Y	N	Y	Y															
White RG and Gumley AI	2009	Y	Y																Y	Y	Y	Y	Y
Bassett M, Sperlinger D and Freeman D	2009	Y	Y											Y	N	Y	Y	Y					
Baker C	1995	Y	Y	Y	Y	Y	N	Y															
Collett N et al.	2016	Y	Y																N	Y	Y	Y	Y
Herz and Melville	1980	Y	Y																Y	Y	Y	N	Y
Sandhu	2013	Y	Y	Y	Y	Y	Y	Y															
Gumley AI et al.	2015	Y	Y						Y	Y	Y	N	Y										

*Y* yes, *N* no

### Quantitative results

#### Overview of quantitative studies

In total, five studies used quantitative methodology—four had cross-sectional descriptive designs [31–34] and one was a randomised control trial (RCT) [20]. Four of these studies recruited patients [20, 31–33], with one retrospective survey recruiting both patients and carers [34]. Our review and synthesis of quantitative studies addressed Research Question 1:

- In studies of fear of relapse, what clinical wellbeing outcomes have been measured and which of these are significantly associated with fear of relapse?

#### Fear of relapse measures

Fear of relapse was assessed (in part) by two psychometric measures, the Fear of Recurrence Scale (FoRSe) and the Mental Health Worries Questionnaire. The FoRSe is a 29-item measure, with items rated against four-point Likert scales (from 1 ‘do not agree’ to 4 ‘agree very much’). It measures early signs of psychosis relapse and has three factors: intrusiveness, awareness, and fear of relapse. The Mental Health Worries Questionnaire is a 25-item measure that uses five-point scales (0–4) and has three factors: preoccupation, conviction, and distress scales. In both measures, higher scores indicate greater endorsement. Two studies used the Mental Health Worries Questionnaire [32, 33] (to assess fear of madness), two used FoRSe [20, 31], and one study [34] asked patients and carers to endorse whether “fear of going crazy” was present prior to a relapse event.

#### Wellbeing measures

No quantitative studies used formal wellbeing outcomes. Therefore, clinical outcome measures were used as a proxy indication of negative wellbeing, as is common within mental health services and research [35]. However, it is important to note that wellbeing and clinical outcome measures are two distinct concepts. Examples of proxy outcome measures included the Beck Anxiety Inventory (BAI) [36], the Penn State Worry Questionnaire, (PSWQ) [37], and the Hospital Anxiety and Depression Scale (HADS) [38]. Full details of the measures are listed in Table 1.

#### Quantitative narrative synthesis results

Within cross-sectional studies, fear of madness was significantly correlated with higher depression (*r* = 0.72) and suicide ideation (*r* = 0.48) [28]. Additionally, fear of madness was associated with lower self-esteem (*r* = − 0.67) [32]. Furthermore, individuals who were more traumatised by their experiences of psychosis and mental health care were more likely to experience fear of relapse [31]. Finally, in the single RCT [20], baseline assessments of 169 participants showed that the intrusions (*r* = 0.50) and fear of relapse (*r* = 0.51) subscales on the FoRSe were significantly associated with greater depression. Taken together, the available studies suggest that fear of relapse is positively and cross-sectionally correlated with experiences of depression and suicide ideation, and negatively associated with self-esteem.

### Qualitative meta-synthesis results

#### Overview of studies and themes

In total, four of the reviewed studies used qualitative methods [39–42]. Key points are summarised in Table 1. Two interview studies explored fear of relapse from the point of view of patients [40, 42], one study used focus groups to explore the point of view of carers [39], and one explored both perspectives [41] using interviews. Our synthesis of qualitative studies addressed Research Questions 2 and 3:

- What do qualitative studies tell us about the meaning of fear of relapse for patients and/or informal caregivers?
- In people with psychosis (and carers), what is the evidence that fear of relapse impacts wellbeing?

The meta-synthesis of extracted qualitative data generated four key themes: fear of losing social and occupational functioning, fear of losing the ability to make autonomous decisions, trauma and re-traumatisation, and the impact of fear of relapse on wellbeing. These are now reported in turn with direct quotes from original manuscripts for transparency.

#### Fear of losing social and occupational functioning

Patients and carers alike described fear of relapse in terms of fears of the patient losing functioning, which could include both social and occupational functioning. For patients in recovery, a potential relapse represents losing everything they have gained.

"Look at everything I've gained and now I'm back to nothing–I felt that I was just going to lose everything I'd achieved."—patient quote [42].

*Participants [carers] with experience of relapse generally described the impact of relapse as a set-back in relation to hope, and social and functional recovery. They used phrases like*, *“back to square one,” “here we go again,” “go backwards,” “fall down,” “backslide,” “repeating,” and “falling off the wagon.” One participant highlighted how relapse impacted her:* “loss of hope because you build so much hope that they’re getting better and getting better and then all of the sudden you have a relapse…”—carer quotes and author analysis (italics) [39]

“Some [patients] feared losing the hard won equanimity they had gained, some feared the torment that accompanied their relapses, and some feared the potential consequences of a relapse on their life”—author analysis [42].

The loss of social and occupational functioning was not just limited to patients. Carers also noted that they had made great efforts to help their loved ones achieve stability and they feared the social and occupational impact that subsequent relapses would have on their own lives.

“Janet [carer pseudonym] thinks that it is most important that he [person they care for] stay in treatment and continue to receive Social Security Disability so that his family does not have to repeat all of their efforts to get him stabilized”—author analysis [41].

#### Fear of losing the ability to make autonomous decisions

A key theme from the meta-synthesis, particularly from studies focussing on the point of view of patients, was fear of losing personal autonomy to make decisions. While this could include fears of being hospitalised involuntarily and not being involved in decisions about healthcare, it also included fears of losing the ability to trust one’s own thoughts and to make everyday decisions.

“She [patient participant] worries about having to go back to the state hospital and starting all over again on different medicines”—author analysis [41].

“Once you realize that you’ve lost track of reality and your mind starts getting to grips with the fact that it was wrong, it was completely wrong for so long, I dunno, you kind of lose your confidence in your own judgment”—patient quote [40].

#### Trauma and re-traumatisation

Trauma associated with prior periods of illness (and fear of re-traumatisation) seemed an important factor in fear of relapse. Trauma narratives dominated Baker’s (1995) interviews, with the author summarising:

“The theme of emotional pain dominated informants’ narratives. Ranging in intensity from sub-acute levels of discomfort to periods of terrible psychic anguish” [42].

An overarching theme of trauma was also apparent from Sandhu et al. Here, a participant described ruminating over their past experiences and feeling deep shame in themselves because of them.

“It’s really hard for me to even deal with the fact that I had an episode. I mean I am starting to come to terms with it now but for a while I could not believe it. It’s more the way I behaved when I had an episode… That makes me feel so, sometimes makes me feel embarrassed about myself.”—patient quote [40].

Additionally, family members felt like trauma pertained not only to the patients but to the caregivers as well, as having the mental health team in the house and watching loved ones being taken away to a psychiatric institution had a big impact [39]: “it was very distressing to me personally… to have the people from the mental health team come there and take him away…it was very upsetting, I could not work for about a week, that’s how upset I was”—carer quote [39].

The meta-synthesis results also covered potential pathways between fear of relapse and negative impact on wellbeing. Across studies, social avoidance was proposed as a potential indirect factor for how fear of relapse results in negative impacts upon wellbeing. While avoidance may make people feel safe initially, as they are protected from the observations (and potential actions) of others, it seems to result in more negative thinking.

“I always think I’m gonna get unwell again and again, that’s maybe another reason why I do not go out, I do not wanna talk to people ‘cause I’m thinking last time, when I had an episode, it was in public that kind of scared me. I’m thinking what if I get unwell again”—patient quote [40].

“Whenever I isolate and I’m alone a lot, then that’s when things go wrong. That’s when my thinking patterns get illogical. That’s when I start thinking and my self-worth, self-esteem goes downhill.”—patient quote [41].

The meta-synthesis suggested that fear of relapse may also have direct negative impacts upon wellbeing. For example, in the focus group study of carers of people who have experienced first episode psychosis, fear of relapse provoked anxiety in carers. When participants were asked what the term relapse meant for them, several responded with expressions such as, “fear, serious fear. Relapse is a scary word for me” [39].

### Mixed-methods summary

In this mixed-methods review, we synthesised quantitative and qualitative findings to address three research questions concerning: the meaning of fear of relapse for patients and carers, associations between fear of relapse and clinical wellbeing outcomes, and evidence fear of relapse causally impacts wellbeing. Taken together, the reviewed studies suggest that fear of relapse is a complex social process involving emotional, social, and behavioural dimensions for both patients and carers. Fear of relapse is associated with lower wellbeing (particularly depression). If assumed to be casual in the way patients and carers describe, this relationship may be driven directly by fear of relapse itself or indirectly via coping strategies such as social avoidance.

### Fear of relapse framework

**Fig. 2:**
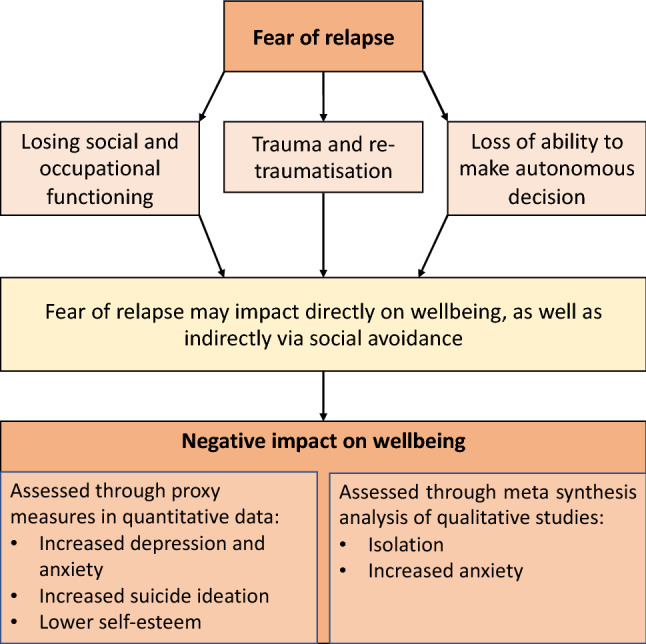
A framework of fear of relapse

The meta-synthesis results suggest the content validity of existing scales could be improved. For example, the FoRSe has an item “The thought of becoming unwell has frightened me” and MHWQ has “I fear I will go mad”. However, the qualitative synthesis outlines several distinct fears of relapse instead of one overarching “fear of relapse”. Therefore, a refined theoretical framework for understanding and measuring fear of relapse should refer to the multiple fears of changes in functioning (including social status) and fear of losing the ability to make autonomous decisions. While MHWQ and FoRSe both address fears of losing the ability to make autonomous decisions about treatment (by assessing concerns about being hospitalised/being locked up forever), the meta-synthesis suggests that these concerns may be part of a broader construct that concerns itself with losing the ability to make autonomous decisions more generally. The framework derived from the mixed-methods review can be seen in Fig. 2.

## Discussion

### Summary of main findings

The purpose of this systematic review was to bring together what is known about fear of relapse and its relationship to wellbeing in schizophrenia, in both quantitative and qualitative peer-reviewed studies. Despite the earliest study from our systematic search being published in 1980, only nine papers met inclusion criteria, indicating that evidence in this area is very limited. Based on our synthesis of available literature, fear of relapse seems a complex experience involving emotional, social, and behavioural dimensions for both patients and carers. The quantitative synthesis revealed two existing psychometric scales (FoRSe and MHWQ) which measure at least some aspects of fear of relapse and demonstrate positive correlations with validated measures of depression [20, 32] and suicide ideation [32]. Additionally, people who have traumatic or distressing experiences of psychosis and of treatment/hospitalisation are significantly more likely to experience fear of relapse [31]. As this research was cross-sectional, it is not possible to comment on causality. However, patients and carers described experiencing negative impacts on wellbeing—apparently emerging from fear of relapse. The meta-synthesis suggested that these negative impacts may stem from social avoidance, wherein people recalled shameful memories about previous episodes of being unwell and retreated socially. Participants stated that isolating themselves was often followed by decreasing self-esteem and thinking becoming illogical. This tentative pathway between fear of relapse and negative outcomes is in line with a previous review’s conclusion that social avoidance is potentially depressogenic in psychosis, because it removes people from the positive and protective benefits of social contact [43]. The proposed Fear of Relapse Framework (Fig. 2) is consistent with research concerning fear of illness recurrence across other health conditions [44]. For example, the theme of “fears of losing functioning” is similar to “fears of disability” subscale in a recent study which validated a fear of relapse scale for people diagnosed with relapsing–remitting multiple sclerosis [45]. Fear of loss of functioning in psychosis is likely not simply an outcome of a relapse itself, but rather a developing awareness that a future relapse will likely create social and occupational losses that are additive to psychosis itself. A recent study [46] suggested that fear of relapse may be higher in patients who have experienced psychosis (if not a schizophrenia diagnosis) compared to those recovering from other mental health problems. Nevertheless, because the between-groups difference effect size was small, fear of relapse may be problematic for people experiencing mental health problems other than psychosis.

### Strengths and limitations

Strengths of the review include a pre-registered protocol, independent screening, data extraction by two reviewers, and the use of appropriate mixed-methods techniques to synthesise and critically appraise the results. However, there are limitations. First, the studies in this review were highly heterogeneous in terms of participant characteristics, data collection methods, and measures of wellbeing and fear of relapse, making synthesis challenging. All studies in our review are potentially skewed towards what the original authors wished to report and the demographics of the original participant samples. For example, the only study which exclusively focussed on carers was drawn from those caring for people who have experienced a first episode of psychosis, specifically [39]. Additionally, patient participants were selected for experiencing paranoid delusions [32] or “relapse proneness” [20] which may limit the validity of findings for people diagnosed with schizophrenia more broadly. Another important limitation is that this review aggregated the perspectives of service users and carers when it is likely they hold diverse perspectives. Additionally, we have not considered the perspective of staff who likely also experience fear of relapse [47, 48].

### Wider context

While the content of this review suggests that relapse is perceived as a negative event, it is worth noting that some antipsychotic discontinuation trials have reported better long-term functional outcomes in individuals stopping antipsychotics, in spite of numerically greater relapse events in those groups [49]. Wunderink and colleagues hypothesised that relapses may present opportunities for learning about self-management which are beneficial for long-term outcomes. Arguably, the prominence of relapse prevention interventions in mental health services may engender fear of relapse within mental health staff. This may make staff hesitant to allow service users to make decisions which are perceived to risk relapse, such as reducing medication [47]. Although relapse is considered common [5], around 20% of patients never relapse [50]. Individuals demonstrate different symptom trajectories, on a spectrum from mild to severe, with some experiencing discrete relapse episodes and others experiencing more continuous psychosis symptoms [51]. Additionally, our proposed fears of relapse might vary across the life stage. Further research on fear of relapse would benefit from engaging with these complexities, with a critical eye on the wider context of relapse as a medicalised and sometimes contentious concept while examining who gets the power define relapse.

### Future research and clinical implications

Clinicians working with people diagnosed with schizophrenia should be aware fear of relapse may be a problem for patients and carers. Fear of relapse may impact on daily life leading to increased isolation and low mood, warranting intervention development. Moreover, people diagnosed with schizophrenia have been observed to experience relapse following exposure to stressful life events [52], and afterwards, may be left with trauma incurred during the episode of psychosis and associated treatment for relapse. These additional problems may mask the impact of the original stressors which remain unaddressed. [53]. From the cancer literature, large effect sizes are found for “third wave” CBT interventions focussed on cultivating attributes of acceptance, compassion, and commitment to valued activities amongst people experiencing fear of cancer recurrence [54]. However, the impact of involuntary or otherwise coercive treatment should be considered in schizophrenia intervention development [55] which may involve trauma focussed interventions [56] on an individual level, and development of general trauma-informed services at an organisational level. Fear of relapse may be a promising therapeutic target for intervention development in people with a diagnosis of schizophrenia that merits further investigation. This work could be underpinned by greater engagement of people with lived experience to inform intervention development and choice of outcomes in line with recent Medical Research Council Guidance [57]. Our findings suggest that existing scales are likely missing some key components the phenomenon. While the meta-synthesis identified potentially useful constructs, which are not currently assessed by either the FoRSe or MHWQ, these were developed from only four heterogenous qualitative studies conducted exclusively in high-income countries. Greater involvement of people with lived experience in the development of improved assessments of fear of relapse in schizophrenia could improve content validity [58]. Further primary research with extensive patient and carer involvement [59] would help identify important fear of relapse constructs which are relevant to people diagnosed with schizophrenia and carers and ensure that the contents of future measures are an adequate reflection of the construct to be measured [60]. None of the reviewed studies used validated assessments of wellbeing, meaning that the impact of fear of relapse on wellbeing was understood through proxy clinical outcome measures. Future research would be greatly enhanced by conducting research with both improved fear of relapse measures and actual validated measurements of wellbeing.

## Conclusions

From the limited literature, fear of relapse appears to be a complex phenomenon with social, emotional, and behavioural components. While further research is needed to explore what fear of relapse means for patients and carers, it also seems pertinent to examine what relapse itself means for patients and carers and how beliefs and experiences intersect with the mental health care system. With existing tools not capturing all aspects of fear of relapse found in our synthesis (e.g., fear of losing social and occupational functioning), collaborative research with strong service user and carer involvement has the greatest potential to enhance understanding of how fear of relapse relates to wellbeing.
